# Single-Cell Profiling of Cells in the Lung of a Patient with Chronic Hypersensitivity Pneumonitis Reveals Inflammatory Niche with Abundant CD39+ T Cells with Functional ATPase Phenotype: A Case Study

**DOI:** 10.3390/ijms241914442

**Published:** 2023-09-22

**Authors:** Tharushi Ayanthika de Silva, Simon Apte, Joanne Voisey, Kirsten Spann, Maxine Tan, Chandima Divithotawela, Daniel Chambers, Brendan O’Sullivan

**Affiliations:** 1 Centre for Genomics and Personalised Health, Faculty of Health, School of Biomedical Sciences, Queensland University of Technology (QUT), Brisbane, QLD 4000, Australia; 2 Queensland Lung Transplant Service, Ground Floor, Clinical Sciences Building, The Prince Charles Hospital, Rode Road, Chermside, Brisbane, QLD 4000, Australia; 3 Facility of Clinical Medicine, The University of Queensland, Brisbane, QLD 4000, Australia; 4 Centre for Immunology and Infection Control, Faculty of Health, School of Biomedical Sciences, Queensland University of Technology (QUT), Brisbane, QLD 4000, Australia

**Keywords:** chronic hypersensitivity pneumonitis, CD39, regulatory T cells, single-cell transcriptomics

## Abstract

This study investigated immune cell characteristics in chronic hypersensitivity pneumonitis (HP), focusing on CD39-expressing cells’ impact on inflammation and tissue remodelling. Lung tissue from an HP patient was analysed using single-cell transcriptomics, flow cytometry, and gene expression profiling. The tissue revealed diverse cell types like macrophages, T cells, fibroblasts, and regulatory T cells (Tregs). CD39-expressing Tregs exhibited heightened ATP hydrolysis capacity and regulatory gene expression. CD39hi cells displayed markers of both Tregs and proinflammatory Th17 cells, suggesting transitional properties. Communication networks involving molecules like SPP1, collagen, CSF1, and IL-1β were identified, hinting at interactions between cell types in HP pathogenesis. This research provides insights into the immune response and cell interactions in chronic HP. CD39-expressing cells dual nature as Tregs and Th17 cells suggests a role in modulating lung inflammation, potentially affecting disease progression. These findings lay the groundwork for further research, underscoring CD39-expressing cells as potential therapeutic targets in HP.

## 1. Introduction

Hypersensitivity pneumonitis (HP) is a common interstitial lung disease, resulting in immunologically driven inflammation in response to inhaled antigens [1]. Animal proteins, fungi, bacteria, and other small-molecular-weighted chemical compounds are known to be common causative agents [2]. HP can be categorised into acute or chronic, based on the nature of exposure, type of antigen, and other host-related and environmental factors. Acute HP generally responds to ‘trigger’ avoidance and symptom management, while chronic HP may be associated with lung fibrosis or non-fibrosis [3]. HP is characterised by an influx of lymphocytes into the alveolar space [4,5]. The disease is driven by both antigen exposure and promoting factors, such as genetics, leading to adverse immune reactions and inflammation [6]. Previous studies have found that interleukin-17 (IL-17) is present in patients with chronic HP, while the levels are low in acute HP and healthy controls [1]. IL-17 is a proinflammatory cytokine secreted by a subset of polarised CD4+ T cells called Th17 cells [7]. IL-17 is known to induce proinflammatory cytokine expression in neighbouring cells and is actively involved in the recruitment and activation of neutrophils and especially in the regulation of neutrophil migration to the lung [8]. In mice, the chronic inhalation of aerosolised antigens increases IL-17-induced lung fibrosis by enhancing pulmonary collagen deposition [9]. IL-17 also promotes airway remodelling via the production of profibrotic IL-6 and IL-11 and matrix metalloproteinase 9 (MMP 9) [8]. Not all HP patients will progress to chronic HP. Many develop mild lymphocytosis in the alveolar space and remain asymptomatic, suggesting a possible development of tolerance to the ‘trigger’ antigen. Regulatory T cells are implicated in this tolerance, but the mechanisms remain unclear [6]. FOXP3+ regulatory T cells (Tregs) are specialised CD4+ T cells with the ability to suppress the proliferation and function of effector T cells and antigen-presenting cells [10,11]. Thus, Tregs play a vital role in limiting immune reactions and lung damage. Tregs express high levels of the interleukin-2 receptor alpha chain (CD25) and the forkhead box protein P3 (FOXP3), a transcription factor controlling development and gene expression signature [12]. In addition, Tregs express ectonucleoside triphosphate diphosphohydrolase-1 (ENTPD1, CD39), lymphocyte activation gene 3 (LAG3), and cytotoxic T-lymphocyte antigen-4 (CTLA4) [13,14]. Impaired Treg function has been reported in chronic HP. Tregs isolated from either the bronchoalveolar lavage (BAL) or blood of chronic HP patients are less suppressive than Tregs from healthy controls [2]. The Tregs of asymptomatic antigen-exposed individuals show an intermediary suppressive activity compared with healthy controls and chronic HP patients [2]. These studies suggest that Tregs suppress disease in asymptomatic subjects but not in chronic HP, where lung inflammation and IL-17 impair Treg function [2]. In support of this view, inflammatory cytokines interleukin-1 (IL-1) and interleukin-6 (IL-6) drive Tregs to produce IL-17 [15,16,17,18,19]. In addition to lymphosuppressive properties, Tregs upregulate the expression of CD39, an enzyme able to deplete adenosine triphosphate (ATP), a mediator of inflammation through purinergic signalling to adenosine [20]. Due to the proinflammatory properties of ATP and immunosuppressive properties of adenosine, the balance between ATP and adenosine is considered to be fundamental in immune homeostasis [21]. In this case report, the cellular composition and functional characteristics of lung tissue in a patient with end-stage hypersensitivity pneumonitis (HP) were investigated. The major cell populations identified were monocyte-derived macrophages, alveolar macrophages, and CD4 and CD8 T cells. This study highlights the proinflammatory and fibrotic characteristics of macrophages and examines the effector and regulatory nature of T cells. Additionally, the study explores the functional activity of CD39-expressing T cells, confirming their higher ATP hydrolysis capacity. These findings contribute to a better understanding of HP and can inform targeted therapeutic approaches.

## 2. Materials and Methods

### 2.1. Isolation of Immune Cells from Explant Tissue

Immune cells were isolated from the explanted lungs of the patient and healthy lung resections as a control group. The tissue digestion mixture was prepared using 0.1 g of collagenase I, dispase, and DNase I in 100 mL of RPMI media. The mixture was then sterile-filtered and maintained at 37 °C. The tissue section was obtained from the explant lungs, minced using scissors, and then incubated with digestion media for one hour at 37 °C on a MACSmix™ tube rotator (Miltenyi Biotec, Bergisch Gladbach, Germany) at maximum speed. Disaggregated cells were sieved using a Corning^®^ cell strainer (Sigma-Aldrich, St. Louis, MO, USA) and spun at 1000 g for five minutes, and the cell pellet was resuspended in media (2% FCS in RPMI) and counted using a Neubauer haemocytometer cell-counting chamber. Cells were cryopreserved in a freezing buffer (final concentration 35% FCS with 7.5% dimethylsulfoxide (DMSO)) for future analysis.

### 2.2. Single-Cell RNA Sequencing

Single-cell sequencing was carried out using the 10× Genomics Chromium platform, using the 3′ RNA v2.0 assay as previously described [22].

### 2.3. Single-Cell Data Analysis

The gene-cell count matrix generated from the 10× Genomics Chromium 3 single-cell analysis was preprocessed by filtering low-quality cells and genes with low expression levels. Gene expression values were normalised using the ‘NormalizeData’ function. Highly variable genes were identified using the ‘FindVariableFeatures’ function, and a subset of these genes was selected for downstream analysis. Principal component analysis (PCA) was performed on the selected genes using the ‘RunPCA’ function. A subset of principal components capturing significant variation was retained. Clustering was performed using the ‘FindNeighbors’ and ‘FindClusters’ functions, and the number of clusters was determined based on different resolutions. Clustering results were visualised using dimensionality reduction techniques such as t-SNE or UMAP. Marker genes for each cluster were identified using the ‘FindMarkers’ function, and clusters were annotated based on marker gene expression patterns and known cell type markers. AZIMUTH package was used for cell annotation in single-cell RNA sequencing (scRNA-seq) data. The package facilitated accurate cell type predictions by training an annotation model on reference datasets and applying it to the target scRNA-seq data. Cell-type predictions were evaluated and validated against known annotations. The package enabled the identification and characterisation of distinct cell types within the scRNA-seq data. CellChat package was used to identify sender and receiver cells involved in cell communication. After preprocessing the scRNA-seq data, CellChat was applied to build a communication atlas and calculate interaction scores. Statistical analysis and visualisation were performed to interpret the results, and biological implications were investigated. CellChat facilitated the identification of cell–cell communication dynamics and regulatory mechanisms in the study.

### 2.4. Flow Cytometric Analysis

Cryopreserved cells (explant cells or healthy lung resection tissue cells) were stained and analysed using flow cytometry. Cryopreserved samples were thawed and spun down, and the cell pellet was resuspended in phosphate-buffered saline (PBS) and 1% foetal calf serum (FCS). Cells were stained with Fixable Viability Stain 620 (BD Biosciences, San Jose, CA, USA) prewarmed to 37 °C and incubated for five minutes at room temperature. Cells were then washed with MACS buffer (MB), 2 mM EDTA, and 0.5% FCS in PBS and incubated with Human TruStain FcX (BioLegend, San Diego, CA, USA) to block Fc receptors (FcR) for 15 min at 4 °C. Cells were washed with MB and resuspended in an antibody master mix to surface antigens for 30 min at 4 °C. The antibody master mix included anti-CD3-AF700 (BioLegend), anti-CD4-BV510 (BioLegend), anti-CD8-APC-H7 (BD Biosciences), anti-CD14—PE-Cy 7 (BD Biosciences), anti-CD25-BV421 (BD Biosciences), anti-CD127-FITC (BD Biosciences), anti-CD39-BV711 (BioLegend), anti-CD73-BV605 (BD Biosciences), anti-CD45RO-BV650 (BD Biosciences), anti-HLA-DR-BV785 (BioLegend), and anti-CD194-PE-Cy 7 (BD Biosciences) at preoptimised working concentrations. After incubation, cells were washed with MB, fixed with a Fix–Perm solution (Transcription Factor Buffer Set, BD Biosciences), and incubated for 30 min at room temperature. Cells were washed and incubated with intracellular staining master mix, including anti-Ki67-PerCp-Cy5.5 (BioLegend), anti-FOXP3-PE (BD Biosciences), and anti-granzyme B-APC (Invitrogen), at optimised concentrations in a Perm–Wash buffer solution (Transcription Factor Buffer Set, BD Biosciences) for 30 min. Flow cytometric analysis of the samples was performed on a BD LSRFortessa™ X-20 Cell Analyser (BD Biosciences), and the data were analysed using KALUZA flow cytometry analysis software version 2.1 (Beckman Coulter, Brea, CA, USA).

### 2.5. Cell-Sorting Experiments

CD39hi and CD39int cells were sorted using flow cytometry (FACSAria II; BD Biosciences) from digested explants from the HP patient. The gating strategy was on live cells with the separation of CD3 + CD4 + CD45RO + CD39hi and CD3 + CD4 + CD45RO + CD39lo, as shown in the Results section. The sorted CD39hi or CD39lo cells (1.5 × 10^5^) were activated in 1 mL of stimulation cocktail containing phorbol myristate acetate (PMA) at 50 ng/mL and ionomycin at 1000 ng/mL for 2.5 h at 37 °C in a six-well plate.

### 2.6. RNA Extraction and cDNA Preparation

RNA was extracted using the commercially available RNeasy Mini Kit by QIAGEN. Manufacturer manufacturer-recommended protocol was followed, and 3 × 10^5^ cells were frozen in 350 µL of RLT buffer provided in the kit. RT-PCR was carried out using the synthesised cDNA at 1 ng/mL. Quantitative real-time PCR (qRT-PCR) was also performed.

### 2.7. TaqMan Gene Expression Assay Probes

TaqMan Gene Expression Assay probes were used to assess expression of B2M, ENTPD1 (CD39), GZMB, IL-10, FOXP-3, IFNγ, HLA-DR, TBX-21, IKZF-2 (Helios), TIGIT, IL-17A, IL-13, LAG-3, IL-21, TNF, JUN-b, KLF-6, PRDM-1 (Blimp-1), EOMES, IL-23, GATA-3, IL-4, PD-1, and HAVCR-2 (TIM-3). Reactions were carried out on a ViiA 7 Real-Time PCR System (Applied Biosystems, San Francisco, CA, USA), and the results were normalised to the reference gene (B2M) using the delta-CT method.

### 2.8. ATP-Assay

ATP was quantified in cell culture media using the commercially available kit ENLITEN^®^ ATP Assay System (Promega, Madison, WI, USA), and 50,000 cells in 2% FCS RPMI media were aliquoted per well in a 96-well plate. To determine if CD39 was driving ATP catalysis, the CD39-specific inhibitor ARL 67156 (Sigma-Aldrich, St. Louis, MO, USA) was used as a control, and the ATP hydrolysis capacity was then calculated per cell.

## 3. Case Presentation

A 40-year-old female presented with 7 years of intractable cough and shortness of breath. There was no significant exposure history. She was a non-smoker and suffered from grass and dust mite allergy. On examination, she had finger clubbing and end-inspiratory fine crackles at both lung bases. A chest CT showed evidence of ground glass opacities, interlobular septal thickening, and focal areas of air trapping in both lung bases, in line with interstitial lung disease suggestive of hypersensitivity pneumonitis. A complex lung function test showed restrictive lung disease with a forced vital capacity of 1.65 L (45% of predicted value) and a total lung capacity of 2.7 L (56% of predicted value). A left-sided video-assisted thoracotomy lung biopsy in late 2017 showed extensive scarring and honeycombing with peribronchial fibrosis and granulomata, which confirmed her diagnosis of hypersensitivity pneumonitis. Her symptoms worsened in early 2018, and she developed a type 1 respiratory failure requiring oxygen. She underwent pulse therapy with an intravenous injection of 1 g of cyclophosphamide and methylprednisolone, and treatment continued with a weaning dose of oral prednisolone. Unfortunately, there was no response to this treatment course, and she underwent bilateral sequential lung transplantation in December 2018.

### 3.1. Single-Cell Sequencing Reveals Proinflammatory Lung Niche in Patient with HP

**Figure 1:**
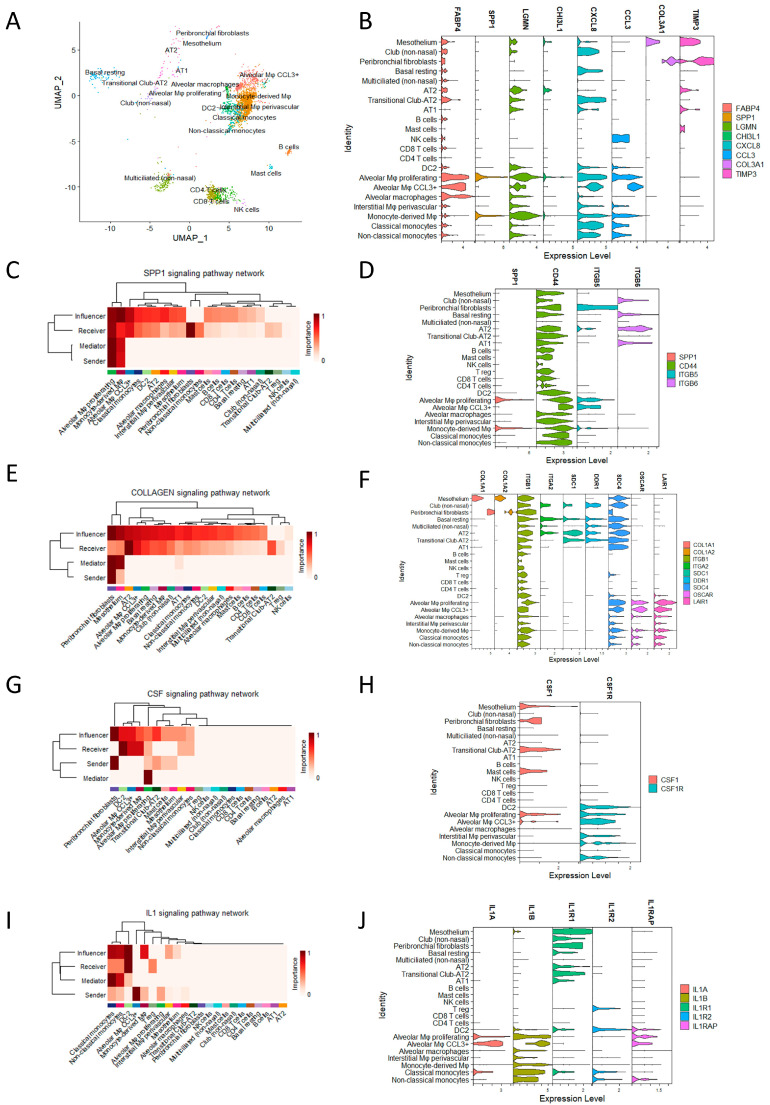
(**A**) Reference-based comparison of cell types isolated from HP explant reveals cell populations; (**B**) stacked VlnPlot showing expression of selected genes implicated in inflammation and fibrosis. CellChat communication networks predicted among various cell types from an HP patient show the strength/importance of cell sender, receiver, mediator, and influencer interactions hierarchically clustered for pathways including SPP1 (**C**), collagen (**E**), CSF (**G**), and IL-1 (**I**). Expression of ligand–receptor pairs for SPP1 (**D**), collagen (**F**), CSF (**H**), and IL-1 (**J**) for cell types are also shown.

Cells digested from explant lung tissue from the HP patient underwent single-cell sequencing. Cells were annotated using the Azimuth R package and the reference Human Lung Cell Atlas (HLCA) (Figure 1A). The main immune cells were monocyte-derived macrophages, alveolar macrophages, and CD4 and CD8 T cells, and parenchymal cells were multiciliated cells and basal resting cells. Cell types and markers specific to each cell type are shown in Figure 1A. Several populations of monocytes–macrophages were identified, including non-classical and classical monocytes, monocyte-derived macrophages, alveolar macrophages (AMs), inflammatory CCL3+ AM, and proliferating AM. Notably, all populations of AMs were characterised by the presence of fatty acid-binding protein 4 (FABP4) (Figure 1B).

Monocyte-derived macrophages exhibited lower levels of FABP4, expressed markers previously associated with fibrotic macrophages, such as secreted phosphoprotein 1/osteopontin (SPP1), legumain (LGMN), and chitinase-3-like protein 1 (CHI3L1) (Figure 1B). These macrophages also displayed proinflammatory characteristics, expressing interleukin-8 (IL-8/CXCL8) and C–C motif chemokine ligand 3 (CCL3) (Figure 1B).

In addition to the monocyte–macrophage populations, other cell types were also identified, including dendritic cells type 2 (DC2), T cells, natural killer cells (NK cells), B cells, mast cells, alveolar type 1 (AT1) and alveolar type 2 (AT2) cells, ciliated cells, club cells, basal resting cells, fibroblasts, and mesothelium. Fibroblasts expressed high levels of collagen and tissue inhibitor of metalloproteinase 3 (TIMP3), which are markers specific to all cell populations (Figure 1B). CellChat was used to deduce the communication networks among cell types from the HP patient’s explant. Sender cells represented cell types expressing ligands, while receiver cells expressed receptors for these ligands. Mediator cells played a crucial role as they expressed both ligands and receptors, acting as bridges between cells to facilitate enhanced signalling. Additionally, influencer cells expressed the highest levels of ligands. Through this methodology, several potential interactions were identified, including CellChat pathways osteopontin (SPP1), colony-stimulating factor (CSF), collagen (COLLAGEN), and interleukin-1 (IL-1).

Considering the SPP1 pathway, monocyte-derived macrophages and proliferating alveolar macrophages (AMs) served as sender cells and expressed CD44, which acted as a receptor for SPP1 (Figure 1C). This finding suggests a potential autocrine role for SPP1 in the proliferation and maintenance of monocyte-derived macrophages. Although SPP1 had the capability to signal multiple cell types through CD44, peribronchial fibroblasts expressing integrin 6 (ITGB6) were identified as the primary receiver cell type for SPP1 (Figure 1D). This signalling pathway is well-known for its involvement in fibrosis, which aligns with the observation of peribronchial fibroblasts expressing abundant collagen type I alpha 1 (COL1A1) and collagen type I alpha 2 (COL1A2).

The collagen pathway was found to mediate signals through integrin receptors for collagen 1 (ITGB1) and syndecan 4 (SDC4), which are ubiquitously expressed. These signals were selectively received by parenchymal cells via integrin subunit alpha 2 (ITGA2), syndecan 1 (SDC1), and discoidin domain receptor 1 (DDR1), and selectively in myeloid cells through the osteoclast-associated receptor (OSCAR) and leukocyte-associated immunoglobulin-like receptor 1 (LAIR1) (Figure 1E,F).

Peribronchial fibroblasts were the main sender cell for the growth factor colony-stimulating factor 1 (CSF1), and myeloid cells expressed the colony-stimulating factor 1 receptor (CSF1R) (Figure 1E,F), suggesting that peribronchial fibroblast crosstalk is important in myeloid cell maintenance through CSF1/CSF1R.

Lastly, myeloid cells, particularly CCL3+ AM, expressed interleukin-1B (IL-1B), which signals via interleukin-1 receptor (IL-1R) expressed by parenchymal cells (Figure 1G,H). This signalling pathway is recognised for its involvement in tissue remodelling and fibrosis and skewing of Treg to Th17.

In conclusion, end-stage chronic HP involves a proinflammatory lung niche characterised by macrophages and prebronchial fibroblasts with significant crosstalk in pathways associated with fibrosis, proliferation, differentiation, and inflammation.

### 3.2. Characterisation of T-Cell Populations within Lung Tissue from HP Patient

T cells play a crucial role in the immune response seen in hypersensitivity pneumonitis (HP). They contribute to the initiation of the inflammatory cascade, the recruitment of other immune cells, the development of immune memory, and the regulation of immune responses. Understanding the role of T cells in HP is essential for developing targeted therapeutic strategies to manage and treat the disease.

**Figure 2:**
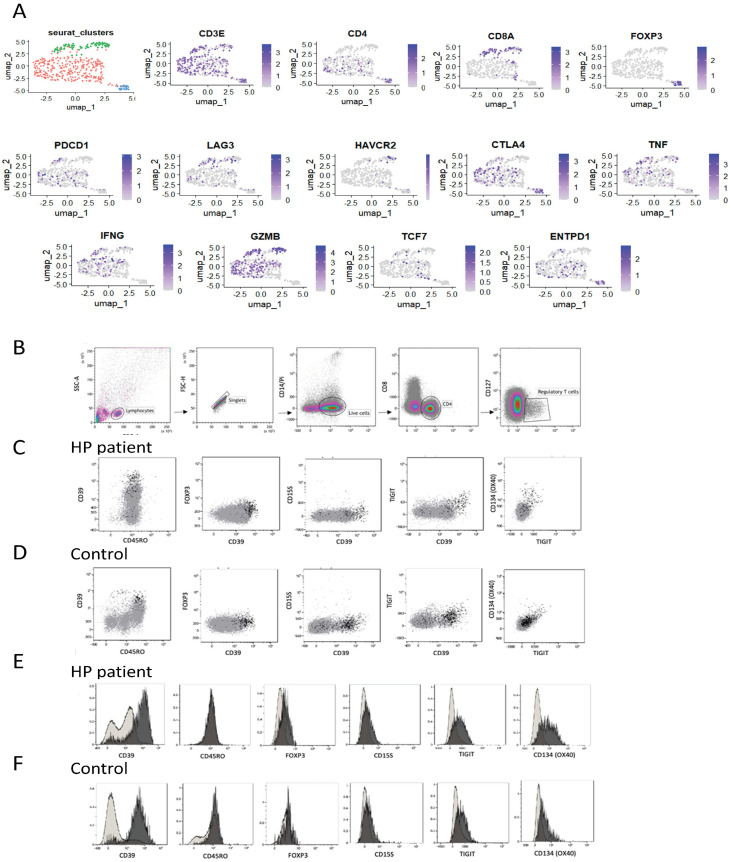
(**A**) UMAP embedding of CD4 T cells with clustering and FeaturePlot showing expression of T-cell genes and inflammatory, cytolytic, and exhaustion markers for T cells; (**B**) representative gating strategy for identification of regulatory lymphocytes. Co-expression CD39 and FoxP3 in regulatory T cells and CD4 T cells (CD4+ T cells shown in grey and CD4 + CD25 + CD127-Tregs in black) for HP explant cells (**C**) and control explant (**D**); differential expression of Treg markers in CD4+ T cells vs. Tregs (CD4+ T cells shown in grey and CD4 + CD25 + CD127-Tregs in black) for HP explant cells (**E**) and control explant (**F**).

In our study on HP, we focused on CD4 and CD8 T cells extracted from single-cell data obtained from the HP patient. These T cells were reclustered, resulting in distinct populations expressing CD4 or CD8. Within the CD4+ population, regulatory T cells (Treg) were identified through the expression of FoxP3 (Figure 2A). Expression analysis revealed that both CD4 and CD8 T cells exhibited the presence of cytolytic molecules, such as granzyme B (GZMB), and inflammatory cytokines like interferon-gamma (IFN-γ) and tumour necrosis factor-alpha (TNF-α), indicating their effector T-cell nature (Figure 2A). However, the CD4 population displayed additional markers associated with chronically activated T cells, including cytotoxic T-lymphocyte-associated protein 4 (CTLA4) and programmed cell death-1 (PD-1). Moreover, CD4 T cells, including Treg, expressed ectonucleoside triphosphate diphosphohydrolase 1 (ENTPD1), also known as CD39 (Figure 2A). To gain deeper insights into these cells, T cells were isolated and characterised from the HP patient’s lung explant based on CD39 expression.

### 3.3. Phenotype of Stimulated CD39hi Cells

**Figure 3:**
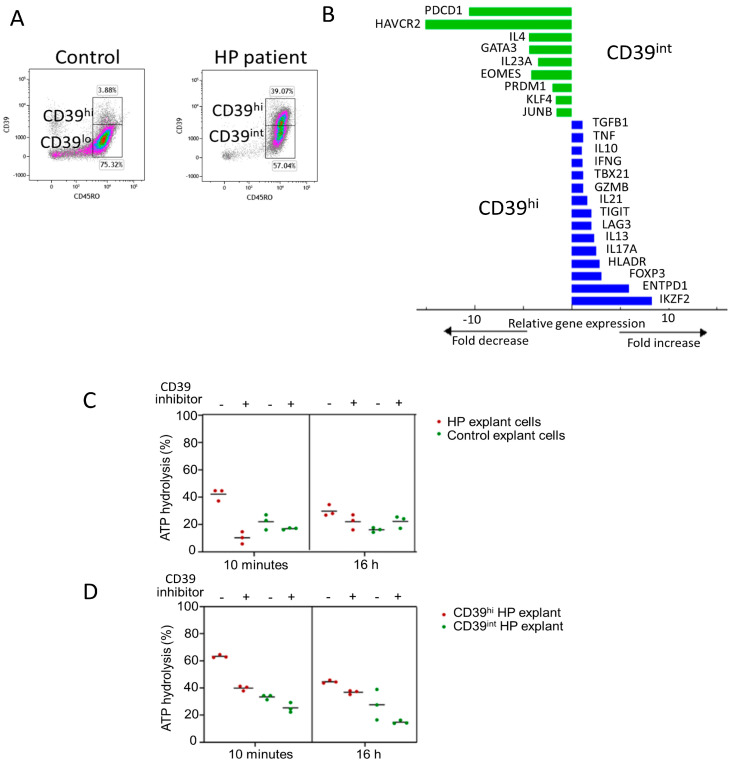
(**A**) Comparison of CD39 expression of control and HP explant CD4 T cells with gating of HP cells used to sort CD39int and CD39high cells; (**B**) quantitative PCR showing expression of genes in flow-sorted CD39int and CD39high cells from HP explant tissue stimulated at 37 °C for 2.5 h with PMA/ionomycin; (**C**) ATPase activity of unsorted HP explant and healthy lung cells incubated +/− CD39-specific inhibitor ARL-67156 for 10 min and 16 h; (**D**) ATPase activity of flow-sorted CD39int and CD39high cells from HP-explant-incubated +/− ARL-67156.

Flow cytometry was used to compare the phenotype of Treg cells in CD4 T cells. CD4, CD25, and low CD127 expression markers were used to identify Treg, as Foxp3 expression was downregulated or challenging to detect in the presence of inflammatory conditions. Tregs were identified by gating on CD3 + CD4 + CD25 + CD127- cells and their expression of Treg-associated markers compared with CD4 T cells (Figure 2B). Tregs exhibited high expression levels of CD39, sialyl Lewis × (CD15S), T-cell immunoreceptor with immunoglobulin and ITIM domain (TIGIT), and tumour necrosis factor receptor superfamily member 4 (OX40) in CD4 T cells (Figure 2C). Tregs were also detected in control lung samples (Figure 2D) but with reduced expression of TIGIT and OX40 compared with the HP patient’s sample (Figure 2E,F). Notably, CD4 T cells with a CD39hi phenotype were enriched in the HP patient’s sample, with 40% of CD39 + CD45RO+ cells in the HP explant compared with 4% in control lung tissue (Figure 3A). CD39hi and CD39int cells were sorted via flow cytometry using digested explants from the HP patient and subjected to 2.5 h of stimulation in culture. qPCR was employed to measure gene expression in the CD39hi cell population relative to the CD39int cell population (gating for sorting shown in Figure 3A). The CD39hi cells exhibited a Treg phenotype with increased expression of Zinc finger protein Helios (IKZF2), ENTPD1 (CD39), FOXP3, and HLADR, compared with the CD39int cells (Figure 3B). Conversely, the CD39int cells expressed markers associated with Th2 cells (GATA3 and IL-4) and memory exhaustion markers (hepatitis A virus cellular receptor 2/T-cell immunoglobulin and mucin-domain containing-3 (HAVCR2/TIM3), and programmed cell death protein-1 (PDCD-1)). Notably, the CD39hi cells also expressed IL-17 and IL-13, indicating potential instability in the Treg phenotype.

In summary, our analysis of CD4 T cells in end-stage HP explants revealed an enrichment of CD4 Th2 cells with a CD39int phenotype. These cells demonstrated Th2 cytokine production capacity and expressed the markers associated with chronically stimulated and exhausted cells (Table 1).

### 3.4. Functional Activity of CD39hi Cells

Previous studies have suggested that the upregulation of CD39 is associated with chronic antigen stimulation in T cells, although its functional effects in this context remain unclear. To confirm the enzymatic activity of CD39, we assessed the conversion of ATP to ADP in cells from HP explants and healthy lung resections. To validate the specificity of ATP hydrolysis, cells were treated with or without the CD39-specific inhibitor, ARL 67156, during both the 10 min and 16 h ATP assays.

At ten minutes, HP cells hydrolysed a higher percentage of ATP (42%) than healthy control tissue (18%). Similarly, at 16 h, HP cells hydrolysed 30% of ATP, while healthy control cells hydrolysed 16%. In the presence of the CD39 inhibitor, ATP hydrolysis was reduced in both tissues (10% HP vs. 17% healthy control at 10 min, and 22% HP vs. 22% healthy control at 16 h) (Figure 3C). These results confirm that CD39 activity is higher in HP cells than in healthy control cells.

Next, we measured ATP hydrolysis in CD3 + CD4 + CD39hi and CD3 + CD4 + CD39int cells sorted from the HP explants. CD39hi cells exhibited a higher percentage of ATP hydrolysis (63.3%) than CD39int cells (33%) (Figure 3D). In the presence of the inhibitor for ten minutes, CD39hi cells hydrolysed 40% ATP, while CD39int cells hydrolysed 25%. After a 16 h incubation with the inhibitor, ATP hydrolysis decreased to 37% in CD39hi cells and 15% in CD39int cells (Figure 3D). These findings confirm that CD4 + CD39hi Tregs have the highest capacity for ATP hydrolysis. Importantly, CD4 + CD39int Th2 memory cells, which are enriched in HP, also exhibit ATP hydrolysis function, albeit to a lesser extent. Since ATP removal is crucial for limiting inflammation, the upregulation of CD39 by chronically stimulated CD4+ T cells may play an important role in reducing lung inflammation.

### 3.5. CD39-Expressing T Cells Enriched in HP and Other ILD

**Figure 4:**
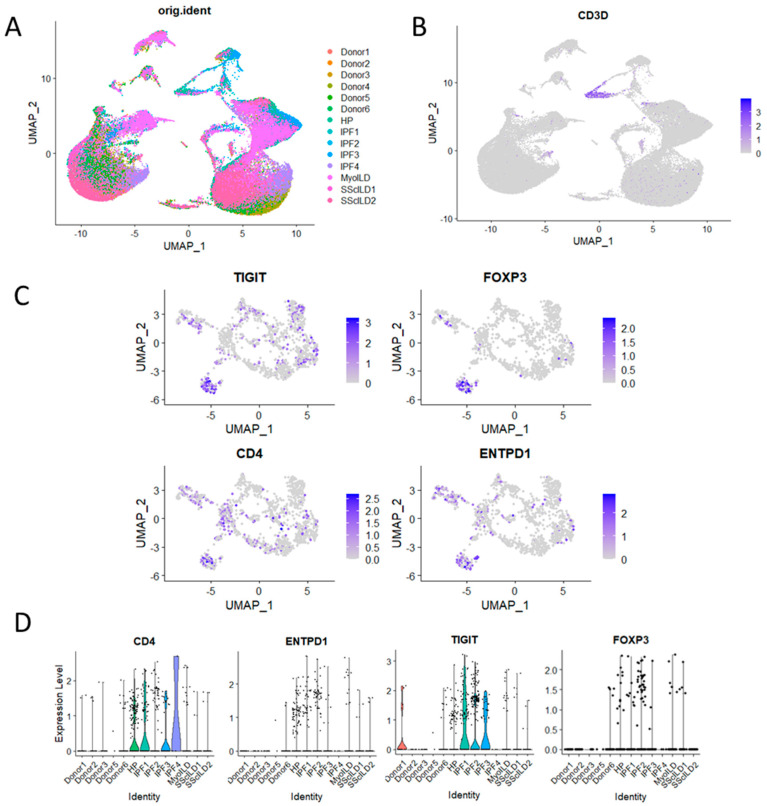
(**A**) Comparison of CD39 expression in control and ILD explant T cells from a published single-cell transcriptomic study (Misharin, http://www.ipfcellatlas.com (accessed on 12 September 2023)); (**B**) UMAP of explant lung cells showing sample clustering; (**C**) T cells identified by CD3D expression were subsetted and analysed for expression of TIGIT, FOXP3, CD4, and ENTPD1; (**D**) violin plot showing mean expression of CD4, ENTPD1/CD39, and TIGIT and FOXP3 grouped by sample type (Donor = control, HP = hypersensitivity pneumonitis, IPF = interstitial pulmonary fibrosis, MyoILD = myositis-associated ILD, and SScILD = systemic sclerosis-associated ILD).

To further validate our findings, we analysed a publicly available single-cell transcriptomic dataset derived from explant tissue samples obtained from control donors and patients with various ILDs (Misharin, http://www.ipfcellatlas.com (accessed on 12 September 2023)) (Figure 4A). Utilising UMAP visualisation, we segregated cells based on disease status, enabling a comprehensive comparison of T-cell populations across different ILDs (Figure 4B,C). After subsetting T cells by CD3D expression, we examined the expression levels of key Treg-associated markers, including TIGIT, FOXP3, CD4, and ENTPD1 (Figure 4D).

Consistent with our case study findings, our analysis revealed that CD4, ENTPD1 (CD39), TIGIT, and FOXP3 exhibited notably higher mean expression levels in patients with hypersensitivity pneumonitis (HP) and interstitial pulmonary fibrosis (IPF) than in control subjects. These findings suggest that the heightened presence of T cells, particularly those expressing CD39, may not be exclusive to HP patients but could extend to individuals with other fibrosing lung diseases such as IPF. This observation underscores the importance of further investigation into the role of T cells in the pathogenesis of these ILDs, offering potential insights into shared mechanisms underlying fibrotic lung disorders.

## 4. Discussion

The results revealed several key findings regarding chronic hypersensitivity pneumonitis (HP). The lung tissue from the HP patient consisted of various cell types, including monocyte-derived macrophages, alveolar macrophages, CD4 and CD8 T cells, multiciliated cells, and basal resting cells. Monocyte-derived macrophages exhibited fibrotic and proinflammatory characteristics, while fibroblasts expressed collagen and TIMP3. Communication networks involving molecules such as osteopontin, colony-stimulating factor, collagen, and interleukin-1 were identified. Peribronchial fibroblasts were identified as the primary receivers of osteopontin signalling, and collagen signalling occurred between parenchymal and myeloid cells. Myeloid cells expressed IL-1β, which signalled to parenchymal cells. CD4 and CD8 T cells displayed both effector and regulatory phenotypes, while CD39-expressing regulatory T (Treg) cells showed increased ATP hydrolysis capacity and gene expression associated with regulatory functions.

The presence of various cell populations, including monocyte-derived macrophages, alveolar macrophages, and CD4 and CD8 T cells, highlights the complex immune response associated with HP. These findings are consistent with previous studies that have implicated these cell types in the pathogenesis of HP [23]. Monocyte-derived macrophages are characterised by fibrotic features, expressing markers such as SPP1, LGMN, and CHI3L1 [24,25]. Fibrotic macrophages have been implicated in tissue remodelling and fibrosis in various lung diseases, including HP [26]. The proinflammatory characteristics exhibited by these macrophages, including the expression of IL-8 and CCL3, further support their involvement in the inflammatory processes observed in HP.

The identification of specific communication networks between different cell types within the HP lung tissue provides valuable insights into the molecular interactions underlying the disease. SPP1 can signal via ITGB5, expressed by fibroblasts and epithelial cells, and through inflammatory–fibrotic macrophages and ITGB6, expressed by basal cells and club cells. SPP1 may play an important role in the proliferation and maintenance of monocyte-derived macrophages and the persistence of these cells in HP.

ITGB5, expressed by fibroblasts and lung epithelial cells, forms the v5 integrin complex with ITGAV. This complex is involved in cell adhesion, migration, and signalling pathways. In lung fibrosis, ITGB5 promotes fibroblast activation and myofibroblast differentiation, leading to excessive collagen production and scar tissue formation. ITGB5 signalling activates profibrotic cytokines and growth factors like TGF-β. Blocking ITGB5 reduces fibroblast activation, collagen deposition, and inflammation, attenuating lung fibrosis in preclinical models.

Additionally, the collagen pathway-mediated signalling between parenchymal cells and myeloid cells, particularly through integrin receptors such as ITGA2, SDC1, and DDR1 [27,28], may play a role in tissue remodelling and fibrosis. The expression of CSF1 by parenchymal cells and its potential signalling to myeloid cells through CSF1R is consistent with previous studies highlighting the role of the CSF1–CSF1R axis in the maintenance of macrophage populations [29]. Moreover, the IL-1β signalling pathway active between myeloid cells and parenchymal cells suggests a role in tissue remodelling and fibrosis, which is supported by previous studies linking IL-1β to these processes [30].

The study findings confirmed the presence of a high proportion of CD4 and CD8 T cells in the explant lung tissue, consistent with an inflammatory response in HP. Specifically, CD4+ T cells expressing high (CD39hi) or intermediate (CD39int) levels of CD39 were identified, with both populations representing memory cells expressing CD45RO+. Gene expression profiling and flow cytometry analysis confirmed that CD4 + CD39hi cells were bona fide regulatory T cells (Tregs), exhibiting high expression of FOXP3, Helios, CD39, and HLA-DR at the gene level, as well as protein expression of FOXP3, CD15S, TIGIT, and OX40. In contrast, CD4 + CD39int cells lacked FOXP3 expression and showed gene expression associated with Th2 cells, as well as genes linked to chronically stimulated and exhausted cells.

Previous research shows that HP is characterised by alveolar lymphocytosis [2] with subacute and chronic HP resulting from amplified T-cell response against inhaled antigens [6]. The dynamics of these cells are not yet completely understood, but increased T-cell migration into the lung, increased localised proliferation, and decreased programmed cell death have been shown in the literature [31]. Among the lymphocytes, we noted that the expression levels of the CD39 were relatively higher in HP explant lung tissue than in normal healthy lung tissue. As CD39 is considered a novel Treg marker, it was of interest to further evaluate the phenotype of these cells to understand their role in the HP lung. From our initial flow cytometry analysis, it was seen that these cells were indeed activated, as most of the cells were positive for HLA-DR, a marker that appears in the late stages of T-cell activation [32]. These cells also expressed CD194, a chemotactic migratory ligand [33]. CD39hiCD45RO+ cells showed a distinct profile compared with CD39intCD45RO+ cells. CD39hiCD45RO+ cells upregulated HLA-DR, IL-17A, IL-13, LAG-3, and IL-21. IL-17A is a proinflammatory cytokine secreted by Th17 cells in response to extracellular bacteria and fungi and is involved in recruiting immune cells to sites of infections [34]. IL-13 is known to be produced by T cells early after activation, typically CD4+ Th2 cells. It has been found to have both proinflammatory and anti-inflammatory properties. IL-13 is involved in macrophage dynamics by enhancing their mobility, functions, and trafficking of macrophages. Additionally, data show that IL-13 can drive fibrosis in the lung in asthma [35] and radiation-induced lung injury [36]. IL-13 might be involved in the allergic conditions that developed in the lung, possibly due to the reactive antigens inhaled by the patient. LAG-3 or lymphocyte activation gene 3 is considered a Treg marker that binds to MHC II of antigen-presenting cells [37]. These cells also expressed higher levels of IL-21, which is considered to play a key role in Th17 cell differentiation [38].

In summary, Tregs stimulated from the HP explant exhibited a gene expression profile of Tregs, TH17, and allergic-IL-13 cells. Tregs and Th17 cells share a common signalling pathway, including TGF-β. Naïve CD4+ T cells differentiate into Th17 cells in the presence of proinflammatory cytokines, such as IL-6 and IL-21 in the presence of TGF-β, and in the absence of proinflammatory conditions, TGF-β drives naïve CD4+ T cells to differentiate into Tregs [39]. Although CD4 + CD39hi cells do not express the hallmark features of Tregs, such as FOXP3, they express higher levels of CD39 and LAG-3, which are also accepted markers of Tregs [13,14]. Since these cells express IL-17A and IL-21, they also display a phenotype of Th17 cells. Overall, we observed dual properties of Tregs and Th17 cells in the novel population of cells expressing high levels of CD39 and CD45RO.

When considering the functionality of CD39 in these cells, they were able to successfully break down extracellular ATP, which notably decreased in the presence of CD39-specific inhibitor ARL-67156, confirming that the ATP hydrolysis observed is typically through the activity of CD39 ectoenzyme. ATP is considered an alarmin, secreted during tissue injury or stress [40]. Previous research has shown that ATP can bind to purinergic receptors and can initiate inflammatory responses via signalling cascades [41]. With the activity of CD39 and CD73, this proinflammatory ATP is broken down into adenosine, which has anti-inflammatory properties [42]. The balance between proinflammatory ATP and anti-inflammatory adenosine is vital in the body and is referred to as the ‘yin and yang’ in immune responses [43]. The cells of interest in this report, with high levels of CD39, may be involved in dampening the inflammatory conditions within the lung that would have resulted from hypersensitivity reactions. These cells may have developed this phenotype to cope with high levels of extracellular ATP released during cell damage from reactive immune cells. It can be hypothesised that these cells may express high levels of CD39 in response to chronic inflammatory conditions of HP lungs, and having expressed CD39, they were able to carry out their enzymatic functions of converting ATP to adenosine in the HP patient.

It can also be speculated that increased CD39 expression in the lung may cause the ATP–adenosine balance to shift towards adenosine, leading to increased extracellular adenosine in the lung microenvironment. Although adenosine is found to be beneficial under acute conditions, reducing inflammation [44,45,46], under chronic conditions, adenosine can cause adverse effects in the lung due to excessive wound healing and tissue repair [47]. Increased adenosine has been reported to be associated with fibrotic lung diseases, such as IPF [48,49,50], asthma [51,52], and COPD [53,54]. It is tempting to suggest that the chronic accumulation of adenosine in the lung tissue due to increased CD39 activity may have led to fibrosis. Although this hypothesis needs further investigation, research in this context would be of value in understanding the complexities in the pathogenesis of HP. It was interesting to observe CD39 cells with a regulatory function expressed IL-17A. These cells may express dual properties, as they could be an intermediary cell population in transition from Tregs to Th17 due to the inflammatory conditions developed from hypersensitivity, particularly IL-1β. Evidence from previous studies shows that Tregs can differentiate into IL-17-producing cells in a colonic microenvironment, psoriasis, colon carcinoma, allergic rhinitis, and polyposis [55,56,57].

To corroborate the findings from the hypersensitivity pneumonitis (HP) case study, we conducted an analysis using a publicly available single-cell transcriptomic dataset accessible via the IPF Cell Atlas (http://www.ipfcellatlas.com). Our analysis revealed elevated mean expression levels of CD4, ENTPD1 (CD39), TIGIT, and FOXP3 in T cells from an independent HP patient, mirroring the observations in the original HP case study. Notably, these findings also extended to patients diagnosed with interstitial pulmonary fibrosis (IPF), underscoring the widespread presence of these molecular signatures within the context of fibrosing lung diseases.

This validation process not only affirms the reliability of the case study’s evidence but also broadens its implications to encompass a wider spectrum of interstitial lung diseases (ILDs), including IPF.

## 5. Conclusions

In conclusion, the results of this study shed light on the cellular composition, communication networks, and functional characteristics of immune cells in HP. The identified fibrotic macrophages, effector T cells, and regulatory T cells provide insights into the immune response and potential pathogenic mechanisms in HP. The elucidation of cellular pathways and interactions, such as SPP1, collagen, CSF1, and IL-1β, offers potential targets for therapeutic interventions. Furthermore, the functional activity of CD39hi cells and their potential role in modulating inflammation highlight the importance of immune regulatory mechanisms in HP. Future studies focusing on these findings may contribute to the development of targeted therapies and improve patient outcomes in HP.

## Figures and Tables

**Table 1:** Flow cytometry and expression studies on CD4 cell types in control and HP lung explants.

Identified Markers	Intended Function	Finding
CD3 + CD4 + CD25 + CD127-	Identification Tregs	
CD3 + CD4 + CD25 + CD127- TIGIT + OX40+	Activated Tregs	Control vs. HP: Increased TIGIT and OX40 expression in HP
CD3 + CD4 + CD39hi	CD4 T cells with high CD39	Control vs. HP: Increased in HP
		CD39hi cells Treg phenotype with increased IKZF2, CD37, FOXP3, and HLADR
CD3 + CD4 + CD39int	CD4 T cells with reduced CD39	CD39int cells Th2 (GATA3 and IL-4) and memory exhaustion markers (HAVCR2/TIM3 and PDCD-1)

## Data Availability

The data that support the findings of this study are available upon request from the corresponding author.
